# Accuracy of a Bedside Heparin Anticoagulation Monitoring Test in Critically Ill Patients

**DOI:** 10.3390/jcdd12100397

**Published:** 2025-10-07

**Authors:** María Teresa Cruces Moreno, Raimundo García del Moral, Manuel Colmenero

**Affiliations:** 1 Intensive Care Medicine, University Hospital Clínico San Cecilio, 18016 Granada, Spain; macol@ugr.es; 2 Ibs.Granada, Instituto de Investigación Biosanitario de Granada, 18016 Granada, Spain

**Keywords:** anticoagulation monitoring, unfractionated heparin, point-of-care devices, agreement, critical care

## Abstract

Anticoagulation therapy with unfractionated heparin (UHF) is a mandatory treatment for many critically ill patients. While the gold standard for monitoring this therapy remains the laboratory-based aPTT (aPTT-lab), the need for immediate results has led to an increase in the development of point-of-care (POC) measurement systems. This study assessed the correlation and agreement between activated clotting time-low range (ACT-LR) and aPTT-POC measurements using aPTT-lab in a cohort of critically ill patients requiring anticoagulation. This prospective cohort study involved patients admitted to the intensive care unit (ICU) who were treated with UFH between January 2022 and January 2024. We performed simultaneous measurements of aPTT-lab, aPTT-POC, and ACT-LR and analyzed 14 samples from healthy volunteers (the control group) to determine the range of normality and mean aPTT-POC. The aPTT-lab value was considered the gold standard measure of coagulation. A poor correlation was observed between ACT-LR and aPTT-lab in the global sample (r = 0.51), which improved slightly when excluding patients with invasive devices (r = 0.61). aPTT-POC showed moderate agreement (bias of 10.4%) but underestimated the aPTT ratio (bias = −0.23), which was similar in patients with and without devices. Agreement was very high in the control group (bias = −0.003). The accuracy of POC anticoagulation monitoring systems is limited in critically ill patients. The aPTT-POC measurements showed better agreement than the ACT-LR measurements. A clinical validation study is needed to adjust for systematic bias in patients with aPTT-POC.

## 1. Introduction

Anticoagulation therapy is widely used in intensive care units (ICUs) and is closely associated with the use of ventricular assist devices (IMPELLA), hemodynamic support with extracorporeal circulation (ECMO), and continuous renal replacement therapy (CRRT). The drug most often used for this treatment is unfractionated heparin (UFH), which requires repeated, rapid monitoring for dose adjustment.

Anticoagulation levels are typically monitored by measuring the activated partial thromboplastin time performed in the clinical laboratory (aPTT-lab) [1]. However, this method has some limitations. Its accuracy may vary depending on the technique used, and sample transport causes delays in obtaining the results, which may compromise measurement precision. The current recommendation for addressing these issues is to standardize aPTT values to calculate an aPTT ratio. This is achieved by normalizing the values measured in heparinized patients to the mean of the normal range in patients not receiving heparin. This standardization also allows for interhospital adjustment by correcting for differences between instruments and reagents. The typical therapeutic range for the aPTT ratio is 1.5–2.5 [2].

The availability of bedside anticoagulation measurement (point-of-care; POC) has important benefits for decision-making [1], primarily for patients at high risk of bleeding, such as those admitted to the ICU. There is a growing trend toward using POC testing, as it provides early results and only requires a low sample volume. This is particularly important for patients who require frequent monitoring of their anticoagulation status throughout the day, which results in a considerable volume of blood being drawn. Werfen Hemochron^®^ provides two ACT-POCs, one for a low anticoagulation range (ACT-LR) and the other for situations requiring higher anticoagulation intensity (ACT+), such as cardiac surgery or interventional procedures.

ACT has been used to monitor anticoagulation in patients with IMPELLA and ECMO ventricular assist devices [3], albeit with a high degree of uncertainty. The ELSO guidelines on anticoagulation in ECMO [2] highlight the lack of correlation between ACT and the aPTT-lab [4] and that the cumulative experience with ACT is limited to cardiac surgery and the catheterization laboratory [5]. ACT-LR or aPTT-POC may be indicated for monitoring and adjustment in the context of low-dose heparin anticoagulation; however, the published evidence on their use in this setting is scarce. Relevant studies have only included patients undergoing cardiac surgery and other hospitalized patients and not patients who are critically ill [6]. The findings of these studies may have limited relevance for ICUs because the UFH doses used in this setting are lower and over a longer duration. Brown E et al.’s 2016 systematic review highlighted a lack of good quality evidence in this patient group [7]. In 2019, Lohith Karigowda et al. conducted the first prospective study on this specific population and observed moderate agreement between whole blood aPTT-POC and aPTT-lab (r: 0.65; 95% range 23.4–28% with a percentage bias of 5%, increasing with test delays above 90 s). However, an analysis of the overall concordance revealed it to be poor, and the authors, therefore, considered the results inaccurate [8]. Therefore, further studies are needed to determine whether new point-of-care anticoagulation devices could be a viable alternative to laboratory testing in these patients. This would offer clear advantages regarding minimizing the volume of blood drawn and increasing the speed at which results are obtained.

This study assessed the correlation and agreement between ACT-LR and aPTT-POC measurements and aPTT-lab results in a cohort of critically ill patients requiring anticoagulation.

## 2. Materials and Methods

### 2.1. Study Design

This concordance and correlation study compared the results obtained using POC tests with those obtained in the clinical laboratory. The variables of interest were aPTT-lab, aPTT-POC, and ACT-LR.

### 2.2. Study Population

From January 2022 to January 2024, we enrolled consecutive patients admitted to a 22-bed ICU who were receiving UFH anticoagulation therapy for any cause, including the use of ventricular-assist devices and extracorporeal circuits (ECMO and CRRT). Patients were excluded if they were less than 18 years old or being treated with other anticoagulants (e.g., low molecular weight heparin, direct thrombin inhibitors). Demographic variables, severity on admission, and indication for anticoagulation were recorded.

### 2.3. Assessment of Laboratory Parameters

For bedside coagulation monitoring, we used the Werfen Hemochron^®^ device (Werfen, Barcelona, Spain) with disposables for determining both ACT-POC and ACT-LR. Blood samples were drawn into a 5 mL syringe in accordance with the usual anticoagulation protocol, at the same time as samples were taken for the aPTT-lab. Thus, three measurements were performed simultaneously, for the aPTT-lab, aPTT-POC, and ACT-LR tests. Non-citrated blood was used for the POC samples. The samples assayed in the laboratory were centrifuged for 10 min to produce platelet-poor plasma. Then, the aPTT-lab was performed on the Werfen ACL TOP 750 system with a reference range of 24–37 s. The reference ranges for normal POC values for the Werfen Hemochron device are 20.6–38.6 s for aPTT-POC and 113–149 s for ACT-LR. The aPTT-lab is considered the gold standard for comparison.

For the control group, 14 samples were taken from patients not receiving heparin to determine their normal range and mean POC measurements. The aPTT-POC ratio was calculated by dividing the result of each patient in the treatment group by the mean result for the control group.

### 2.4. Statistical Analysis

The R-Commander software combined with the R-Commander EZR plugin was used to calculate the sample size. To compare two proportions with 10% disagreement (90% probability of agreement vs. 80%) between them, 106 measurements were required.

Data were imported from an Excel spreadsheet and analyzed using the statistical package R-Commander 2.7-2 (R v 4.2.0). Categorical variables were reported as frequencies and percentages and continuous variables as means (standard deviation) or medians (inter-quartile ranges).

Comparisons between aPTT-lab and ACT were performed using scatter plots and Spearman’s correlation coefficient (r). Correlations were interpreted as either low (r < 0.6), moderate (r between 0.6 and 0.8), or strong (r > 0.8) [9]. The concordance analysis of the aPTT-Lab and aPTT-POC measurements was performed using the Bland–Altman method and by constructing disagreement–survival plots [9]. The comparison with aPTT values was performed using the normalized ratio. The Bland–Altman method assesses the degree of agreement between two quantitative measures by constructing a graph on which the ordinate axis represents the difference between the measurements and the abscissa axis represents their mean. A perfect agreement produces a line parallel to the abscissa axis at point 0. The resulting scatterplot enables visual and numerical estimation of the agreement by ascertaining the mean difference between the measurements and the upper and lower cut-off points (±2 dt).

If there is good agreement, the mean difference between the measurements will be close to zero and the point cloud will be between ±2 dt cut-off points. Following the recommendations of the UK’s National External Quality Assessment Scheme (NEQAS) and the USA’s Clinical Laboratory Improvement Amendments (CLIA), we calculated the plot difference as a percentage, considering the POC determination accurate if it is within 10–15% [8].

The disagreement–survival curves allow for the assessment of the percentage of disagreement between two estimates for different cutoff points. The percentage of agreement at the absolute value of the measurement difference was calculated from the cut-off point of the curve to the upper value of the ordinate axis. To assess the direction of bias and evaluate the influence of different factors, we performed a stratified analysis in accordance with the method described by Llorca and Delgado-Rodríguez [10].

### 2.5. Ethics

This study was approved by the institutional ethics committee of the Hospital Universitario Clínico San Cecilio de Granada (No. 1970-N-21). The requirement for written informed consent was waived.

## 3. Results

We included 30 patients with 242 anticoagulated measurements and 14 patients without anticoagulation as the control group. Patient characteristics are summarized in Table 1. An intermediate analysis was performed on the first 150 measurements. Given the detection of a low correlation between ACT-LR and aPTT-lab, the study was continued until 30 patients had been recruited, excluding ACT-LR measurements in subsequent samples

### 3.1. Description of the Group

**Figure 1:**
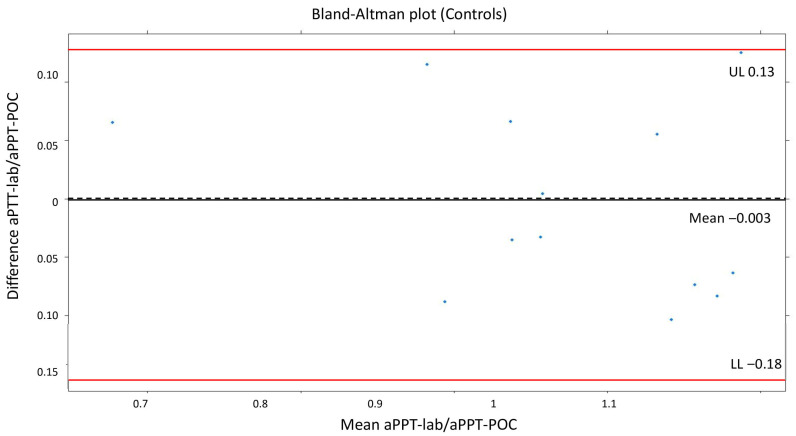
Bland–Altman plot (controls). The mean difference in the aPTT-POC/aPTT-lab ratio was −0.003 (dt 0.08).

The means of the measurements in the group not receiving UFH were as follows: ACT-LR 143 s (dt 10.5, min 130–max 162), aPTT-lab 25.1 s (dt 2.8, min 18.5–max 30), ratio 0.96 (dt 0.11, min 0.71–max 1.15), and aPTT-POC 39.4 s (dt5.3; min 26.1–max 45.1). The mean difference in the aPTT-POC/aPTT-lab ratio was −0.003 (dt 0.08). The Bland–Altman plot for PTT-POC is shown in Figure 1. A good agreement between aPTT-POC/aPTT-lab was noted visually, and the mean of the difference was very close to 0. The cutoff point for 90% agreement is 0.1 for the aPTT-POC ratio. The correlation between ACT-LR and aPPT-lab was significant but moderate (r = 0.7, *p* = 0.01).

### 3.2. Description of Patients Treated with Heparin

#### 3.2.1. ACT-LR and aPTT-lab Correlation

**Figure 2:**
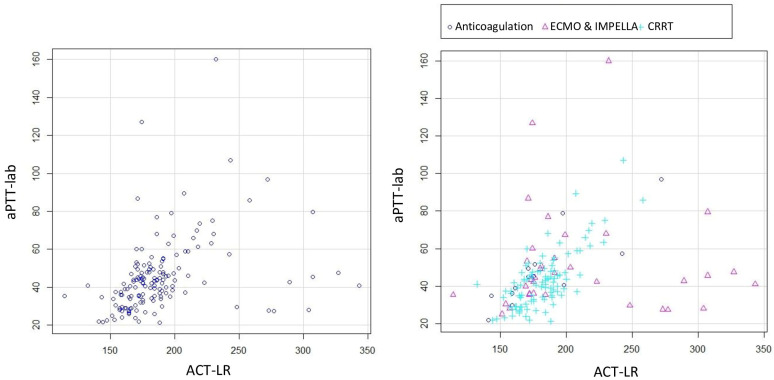
Scatter plots for ACT-LR and aPTT-lab. All the patients (**left**) and stratified by therapy (**right**).

The mean ACT-LR was 185 s (dt 37, min 114–max 343). Figure 2 presents the scatter plots for ACT-LR and aPTT-lab, both overall and stratified by therapy. A better relationship was observed by visual inspection in the group of patients without ventricular-assist devices. The correlation for the total number of measurements is low with a correlation coefficient of 0.51. The correlation improved (r = 0.61) when excluding patients with ventricular-assist devices, although it remained low

#### 3.2.2. Correlation Between aPTT-lab and aPTT-POC

**Figure 3:**
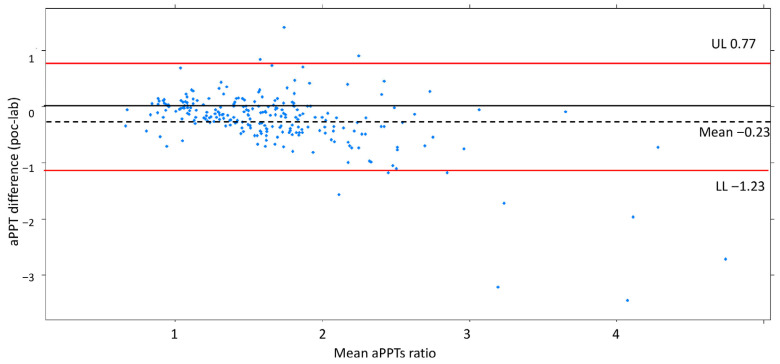
Bland–Altman plot showing a reasonable agreement by ±2dt. The negative bias indicates aPTT-lab is underestimated compared to aPTT-POC. Shows a good degree of agreement between the measurements for 2dt (continuous line).

**Figure 4:**
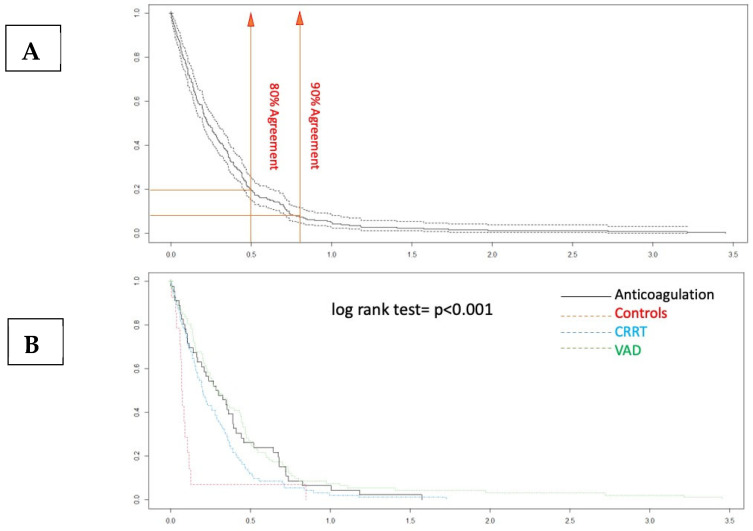
Disagreement–survival plots. (**A**) The cutoff points for 80% and 90% agreement are 0.48 and 0.71 for the absolute difference in ratios. (**B**) In the stratified disagreement–survival analysis, the agreement is similar for the ventricular assist device, renal replacement therapy, and anticoagulation without devices groups.

The mean for aPTT-lab was 44.3 s (dt 19.3; min 18.5–max 160), with a ratio of 1.67 (dt 0.72), and for aPTT-POC it was 59.2 s (dt 20.2, min 19.8–max 146), with a ratio of 1.37 (dt 0.59). The mean ratio difference was 0.23 (UL 0.77, LL −1.23). The Bland–Altman and disagreement survival plots are depicted in Figure 3 and Figure 4.

**Figure 5:**
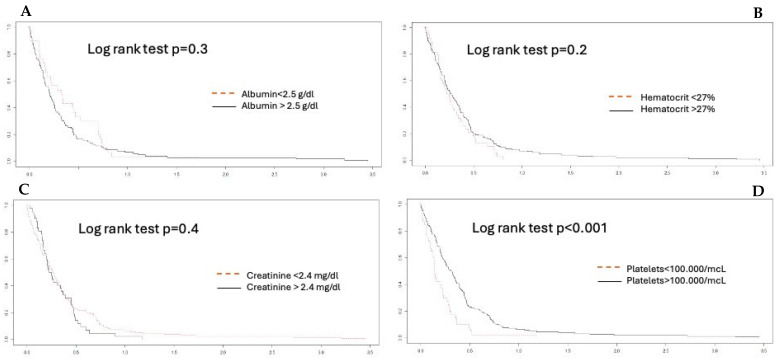
Agreement stratified analysis. (**A**) albumin; (**B**) hematocrit; (**C**) creatinine; (**D**) platelets. A platelet count lower than 100,000 mcl is significant.

The Bland–Altman plot demonstrates a good degree of agreement between the measurement methods within 2dt (continuous line, Figure 3). The negative bias value indicates that the aPTT-lab is underestimated by the POC determination. The mean plot difference as a percentage is 10.8% (limits of agreement 61.76; −40.96). To accurately identify the points of agreement at 80% and 90%, disagreement–survival plots were constructed (Figure 4). The cutoff points for 80% and 90% agreement are 0.48 and 0.71 for the absolute difference in ratios. The stratified disagreement–survival analysis revealed a similar level of agreement between the ventricular assist device, renal replacement therapy, and anticoagulation without devices groups. To assess the influence of potential confounders on bedside whole blood samples, we performed a stratified analysis using albumin (≥2.5), platelet count (≥106), hematocrit (≥27%), and serum creatinine (≥2.4 mg/dL). The only variable related to concordance was the platelet count (Figure 5).

### 3.3. Analysis of Outliers

The Bland–Altman plot (Figure 3) illustrates worse concordance between the measurements at high-ratio values. Three extreme measurements with differences between aPTT-lab and aPTT-POC were detected. The values correspond to measurements in patients with excessive anticoagulation (aPTT-lab ratio greater than 3). In all cases, aPTT-lab was underestimated. Excessive anticoagulation was detected with bedside measurement in two patients.

## 4. Discussion

This study found that aPTT-POC and ACT-LR measurements had moderate accuracy for monitoring POC anticoagulation in critically ill patients treated with intravenous heparin. This result contrasts with the near-perfect accuracy in the healthy volunteer group. The discrepancy is due to the presence of various factors in critically ill patients that distort their results.

Anticoagulation monitoring is essential in critically ill patients receiving intravenous heparinization to avoid complications due to under- or overdosing, especially in those with devices. There remains a lack of standardization in monitoring, as highlighted by the results of the latest ELSO 2024 survey of European centers involved in the care of adults receiving mechanical cardiocirculatory support. Of the respondents, 72.4% adhered to locally established protocols, with laboratory-measured aPTT monitoring predominating (43.1% using IMPELLA and 32.9% in ECMO patients). In the remaining cases, considerable heterogeneity was observed between centers [10,11].

Despite the known benefits of intravenous heparinization, it can have an unpredict-table dose–response effect in critically ill patients, making its use challenging. This group is characterized by a high risk of both bleeding (the most frequent and feared complication in patients with devices) and thrombotic events. POC systems, if determined to be accurate, will help with the rapid adjustment of UFH doses, thereby improving patient care by reducing the delay in obtaining results, which is inherent in sample transport and processing. This will facilitate fast and accurate decision-making. However, although POC testing is currently available, studies assessing the accuracy and validity of its use with ECMO [2] are very limited, as recognized in the ELSO 2021 anticoagulation guidelines.

The evidence regarding the accuracy of POC devices in critically ill patients receiving intravenous heparinization is limited and conflicting. Studies have used different POC devices (Hemochron 801, Hemochron Junior, Hemochron Response, Hemochron Signature Elite, etc., Werfen, Barcelona, Spain) and different case mixes (ICU or non-ICU patients), with or without therapeutic anticoagulation using intravenous heparinization. Our study used the Hemochron Werfen^®^ device, making it challenging to compare our results with those of studies using other tools.

Among the coagulation parameters measured bedside, ACT is the most widely used. The correlation between ACT and aPTT-lab is poor [6,7], with aPTT-lab [8] considered superior. The limitations of ACT in this patient population have been described extensively, with its results being insensitive to various factors (platelet alteration, fibrinogen levels, etc. [12]). This has led to a progressive shift from the use of ACT to aPTT-lab, which is clearly reflected in the ELSO surveys (2013 and 2021 [13,14]). Adding the ACT-LR test to the range of tools used for low-dose heparin monitoring was an attempt to overcome the problems associated with ACT. However, its accuracy has also been questioned, with up to 22% of its results being classified as incorrect during vascular surgery with low-dose intravenous heparinization [15]. Atallah et al. [16] found a low correlation between paired samples of ACT-LR and aPTT in ECMO patients (r = 0.41), as well as with the dose of heparin administered (r = 0.11–0.14). These data were also reproduced in patients who underwent ECMO-PCR with even lower correlations [17].

Our study found a stronger correlation for the ACT-LR in the control group (r = 0.7) than in those treated with intravenous heparin. This may be because critically ill patients have various factors, such as alterations in hematocrit and platelets, that can influence the analysis using whole blood samples standardized to normal values. This potential bias is even more evident in patients treated with ventricular support devices who are routinely subjected to thrombopenia, transfusions, and hemodilution. In medically anticoagulated patients, the correlation has been observed to be higher (r = 0.7), and, in our cohort, it was even higher when including both the control group and the medically anticoagulated patients (r = 0.8), supporting the potential influence of these external factors on the validity of the ACT-LR.

Published studies on the validity of aPTT-POC as a substitute for laboratory tests have not produced satisfactory results. Gauss et al. demonstrated poor concordance in patients with acute bleeding requiring transfusion [18]. This study did not reference the normality value used for calculating the ratio between the POC and the laboratory samples. They used Hemochron Signature Elite for this purpose. They defined acceptable agreement as no more than 5% of the aPTT-POC results, differing from the aPTT-lab results by more than 20%. Their results showed that a total of 89% of the aPTT-POC measurements were out of range. It is assumed that bleeding is associated with rapid changes in hematocrit and platelet counts and other factors, including fluid therapy and transfusion [19]. In our case, after considering these confounding factors, we observed that the presence of thrombopenia < 100,000/µL modified the concordance significantly (Figure 5).

The first study to assess the accuracy of aPTT-POC in ICU patients was published in 2019 [8]. This prospective study included patients requiring all-cause anticoagulation in the ICU (with a mean APACHE II severity score of 18 points). The main indications for UFH infusion were pulmonary embolism and atrial fibrillation. Laboratory and POC values in both whole blood and citrated blood were compared according to whether the aPTT was less or more than 90 s. The limits of agreement were wider, and the bias was greater with aPTT POC obtained using citrated blood than with that obtained using whole blood. Agreement was better at aPTT POC values below 90 s, and bias increased with increasing aPTT POC values. The results showed that adjusting the UFH dose based on the aPTT-POC measurement resulted in more patients receiving inadequate rather than excessive doses. These findings highlighted the need for future studies to establish an adequate aPTT range. Our study found that the aPTT-POC measurements underestimated the laboratory values (Figure 3). However, it should be noted that the agreement is better when the aPTT ratio is in the 1.5–2 range, which is the usual target aPTT range for anticoagulation with UFH. The lowest agreement was observed at high anticoagulation values.

Studies on ICU patients with devices, most frequently in the context of ECMO use, have not demonstrated adequate accuracy. Yuan-Teng et al. [20] found low concordance between both tests regarding both the time and ratio. aPTT values between 23.2 and 38.7 s, which were obtained from Werfen, were considered normal. Our cohort of healthy volunteers exhibited higher values of normality; therefore, there was greater adjustment of the ratios, which would explain the superior concordance we observed. In another study conducted in patients receiving V-V ECMO support for ARDS secondary to SARS-CoV-2, low concordance was observed between aPTT-POC and aPTT-lab, with increased concordance when the results were normalized to platelet and creatinine counts. The authors believe that platelets and renal function may influence the outcome of whole blood POC testing [21].

We identified only one study with satisfactory agreement between laboratory values and those of the bedside test, observing a correlation of r = 0.867 (*p* < 0.001; with HEMOCHRON Jr., International Technidyne Corporation (ITC), Edison, NJ). This study was conducted in the context of heparinization for cardiovascular surgery and during the operating room stay [5], which differs considerably from our population of critically ill patients.

Methodological differences may partly explain the poor agreement between the POC measurements and the aPTT-lab. The reagents used in POC testing are phospholipids and kaolin, whereas aPTT-lab uses silicon dioxide, ellagic acid, calcium, and phospholipids. There are also differences in the principles used for time-to-clot detection (photoelectric versus viscoelasticity) [8]. In addition, the POC method uses whole blood, whereas the laboratory method uses plasma devoid of white blood cells, red blood cells, and platelets. This processing of the blood prior to aPTT measurement may eliminate some of the variation and individual influences, particularly those related to platelet count, and therefore may account for some of the discrepancy in the results. POC assays are derived from whole blood by using an algorithm to calculate aPTT-POC. The results are theoretically calibrated to normal hematocrit and platelet counts. Some manufacturers’ specifications for POC measurements recommend excluding blood samples with a hematocrit < 20% from measurements of aPTTT-POC, as the optical density of the sample exceeds the detection range. Different coagulation activators produce different results [22,23].

Our study provides important data that builds upon the findings of previous research. First, we used the normalized aPTT ratio for our comparisons, which was obtained by dividing each patient’s aPPT-POC test result with respect to the means of the control group’s results and the result obtained in the hematology laboratory. These data are particularly relevant since in our cohort the normality values are 22–31 s for the aPTT-lab and 39.7 s (dt 5.3; min 26–max 45.1) for the aPTT-POC (control group), which are slightly different from those supplied by the manufacturer. Second, this is a prospective study (most existing studies are retrospective and use databases) that includes a heterogeneous cohort encompassing the various types of patients that can be found in the ICU, as well as the different support therapies that are currently the main indications for UFH anticoagulation. Previous studies have tested the measurements in groups of patients subject to greater bias due to the presence of thrombopenia, hemodilution, and multiple transfusions, such as acute hemorrhage and ECMO treatment. Finally, we analyzed more samples than the recommended minimum sample size of 40.

The current study has several limitations. It was a single-center study with a limited number of patients. Some important variables, such as anti-Xa levels and anticoagulation factors, were not available. It was also not possible to monitor the concentration of heparin and its relationship with POC measurements, because it was not available at our center.

## 5. Conclusions

Among the two POC tests evaluated, aPTT-POC exhibited the best agreement with the laboratory results. Although its concordance is only moderate and requires adjustments in its application, it could be considered for anticoagulation monitoring in critically ill patients with and without devices. Although point-of-care anticoagulation monitoring systems have significant advantages in critical patients, their current accuracy precludes their clinical use without further validation studies.

## Figures and Tables

**Table 1:** Patient characteristics. Number of patients (N); number of samples (n).

Characteristics N = 30		n = 256	%
Ages (mean)	61.3 (dt 12.3)		
Sex	Male 17		56.6%
	Female 13		43.4%
Indication of treatment	Need for anticoagulation	46	17.9%
	ECMO	34	13.3%
	CRRT	102	39.8%
	IMPELLA	60	23.4%
	Control patients	14	5.6%
Hematocrit (mean)	31 (dt 5.3)		
Creatinine (mean)	1.84 (dt 1.28)		
Albumin (mean)	2.76 (dt 0.38)		
Platelets (median)	183,000 (IQR 108,000, min 40,000, max 560,000)		

## Data Availability

The data supporting the findings of this study are available from the corresponding author upon reasonable request. This article is part of the first author’s (María Teresa Cruces Moreno) doctoral thesis, which is being undertaken as part of the PhD program in Clinical Medicine and Public Health at the University of Granada.

## Abbreviations

The following abbreviations are used in this manuscript:

UHF	Unfractionated heparin
aPTT-Lab	Laboratory aPTT
POC	Point of care
ACT-LR	Activated clotting time-low range
ICU	Intensive care unit
CRRT	Continuous renal replacement therapy
ECMO	Extracorporeal circulation
NEQAS	National External Quality Assessment Scheme
CLIA	Improvement Amendments in the USA
